# Validation of a novel testing machine for the investigation of the biomechanical properties of lumbar vertebrae in an osteoporotic rat model

**DOI:** 10.1186/s13018-023-03751-3

**Published:** 2023-03-31

**Authors:** G. A. Mackert, M. Harder, H. Harhaus, M. Schulte, U. Trinler, S. Jaeger, U. Kneser, L. Harhaus, C. Wölfl

**Affiliations:** 1 Department of Hand-, Plastic and Reconstructive Surgery, Burn Center, Department of Plastic Surgery of the University of Heidelberg, BG Trauma Center, Ludwigshafen, Germany; 2 Department of Orthopedics and Trauma Surgery, GRN Clinic Weinheim, Weinheim, Germany; 3 Technical and Medical Devices Development and Invention Center, Remscheid, Germany; 4 Laboratory of Biomechanics and Implant Research, Department of Orthopedic Surgery, Heidelberg University Hospital, Heidelberg, Germany; 5 Department of Orthopedics and Trauma Surgery, Marienhausklinikum Neuwied, Teaching Hospital of the Johannes Gutenberg-University, Mainz, Germany

**Keywords:** Biomechanics, Bone, Spine, Small animal model, Rat vertebrae, Rat vertebral body, Lumbar vertebrae, Osteoporosis, Biomechanical analysis, Vertebral body, Compression testing, Axial force application, Vertebral fracture

## Abstract

**Background:**

For the investigation of the biomechanical properties of bone, various testing devices have been described. However, only a limited number have been developed to test the vertebral body of small animals. The aim of this study was to develop and validate a new bone testing device, which investigates the different biomechanical properties in small-animal vertebrae as a whole, three-dimensional unit, respecting its anatomical structure.

**Methods:**

Thirty-five twelve-week-old female Sprague Dawley rats were utilized. Group 1 was composed of 17 rats with a normal bone metabolism without osteoporosis, while Group 2 consisted of 18 rats with manifest osteoporosis, 8 weeks after ovariectomy. The 5th lumbar vertebra of each animal was tested using the new bone testing device. This device has the ability to be adjusted to the slanted nature of each individual vertebral body and fix the vertebra in a natural position to allow for a non-dislocating axial force application. The device is designed to respect the anatomical three-dimensional shape of the vertebral body, thus avoiding the application of non-anatomic, non-physiological forces and thus preventing a distortion of the biomechanical testing results. The parameters investigated were stiffness, yield load, maximum load and failure load, and the results were compared to current literature values.

**Results:**

The conduction of the biomechanical bone testing of the vertebral bodies with the new device was conductible without any instances of dislocation of the vertebrae or machine malfunctions. Significant differences were found for stiffness, maximum load and failure load between groups, with a lower value in the osteoporotic rats in each parameter tested. The yield load was also lower in the osteoporotic group, however not significantly. The values achieved correlate with those in current literature.

**Conclusions:**

This study demonstrates that the newly developed testing machine is easy to handle and produces valid data sets for testing biomechanical bone parameters of whole vertebral bodies in an established small animal model. Therefore, it can be utilized, also as reference data, to test different structural properties and changes in vertebral bone, for example, in different metabolic settings or under the influence of different pharmaceutical entities in further studies.

## Background

Biomechanical testing of the structural properties of bone is a vital aspect of bone research. Tensile strength tests and compression tests, torsional strength testing and three- and four-point bending and breaking tests are some examples of the variety of biomechanical testing options for bone available [1–5]. Although various test protocols for vertebral bone and vertebral device testing, such as cages or pedicle screws, have been established, only few of these use a model assessing the lumbar vertebral body as a whole [6, 7]. Yao et al. for example, used a mechanical testing model using only a 4-mm cancellous portion of a rat lumbar vertebra by cutting away the cortical portion, instead of subjecting the whole lumbar vertebra to compression testing [8]. Rincon-Kohli et al. used a similar technique utilizing only the trabecular portion of human vertebral bodies by cutting out a sample of 8 mm by 10 mm, however by doing so, not utilizing the vertebra as a whole, functional unit [9]. Therefore, even though many different attempts at investigating the biomechanical properties of lumbar vertebrae have been undertaken, there has to our knowledge not been a description of a machine that tests vertebrae of small animals as a whole, functional unit respecting its exact anatomy and physiology, which was the cause for the development of this new testing device for small animal vertebrae in this study.

Vertebral bodies consist of mainly cancellous bone, with cortical bone making up the circumferential edge of the vertebra as well as the top and base plates. Bone, especially cancellous bone, is sensitive to changes in bone–mineral homeostasis and is therefore directly affected by bone maladies such as osteoporosis. This leads to a decrease in trabecular structure which in turn diminishes bone strength and leaves the bone structure weaker and predisposed to fracture events. Therefore, the spine, with its central role in supporting the body in all movements and posture, is one of the most important areas of clinical interest concerning osteoporosis and its consequences [10, 11].

The aim of this study was to develop, describe and validate a novel device for the biomechanical testing of vertebral bodies of the lumbar spine for small animal models as a whole, three-dimensional unit in order to provide a means to test small animal vertebrae, increase the accuracy of the results of the force situation present in the process of fracturing regarding the whole vertebra as a functional unit and to provide a reference data set for the biomechanical properties of lumbar rat vertebrae. The new device was then validated using an established animal model. Using the new testing machine, the biomechanical parameters of the vertebral bodies of osteoporotic bone were compared to the biomechanical properties of vertebral bodies of non-osteoporotic bone. The results were additionally evaluated with regard to the literature.

## Methods

### Study design and specimen preparation

The design of the study was established together with the Institute of Medical Statistics and Documentation of the University of Mannheim. The study design was approved by the ethical committee of the local governmental authorities (Deutsches Landesuntersuchungsamt Koblenz, Rheinland-Pfalz) and complied with all their animal research guidelines (animal research ethical approval number 23177-07/G14-7-056). Further, the animal research conducted in the study adheres to the ARRIVE guidelines as outlined by the National Centre of the Replacement Refinement and Reduction of animal Research.

Thirty-five three-month-old female Sprague–Dawley rats were utilized. Seventeen animals were Sham-ovariectomized, representing the bone-healthy, non-osteoporotic group (Group 1). Eighteen of them were ovariectomized and developed manifest osteoporosis (Group 2) after 8 weeks [12]. The animals were housed in standard Makrolon M-IV cages (Techniplast, Pontremoli, Italy) in non-changing groups of 3–5 animals per cage. A 12-h light and dark photoregimen was implemented at a constant temperature of 22 ± 2 °C and non-changing humidity of 50 ± 10%. The food consisted of soy-, estrogen- and phytoestrogen free food pellets (Sniff, Spezialdiäten GmbH, Soest, Germany). Water was freely available.

The euthanization was conducted via an intraperitoneal overdose of Narcoren® at the end of week 8 after ovariectomy and SHAM-ovariectomy. The fifth lumbar vertebra from all 35 animals was harvested for the testing procedure and meticulously freed of any adherent soft tissue. Then the lumbar vertebral bodies were frozen at − 20° centigrade immediately following harvesting. In advance to the biomechanical testing, x-rays were done in anterior–posterior, lateral and axial view to exclude preexisting fractures or damage caused by the preparation of the specimen.

### New developed testing device

**Fig. 1:**
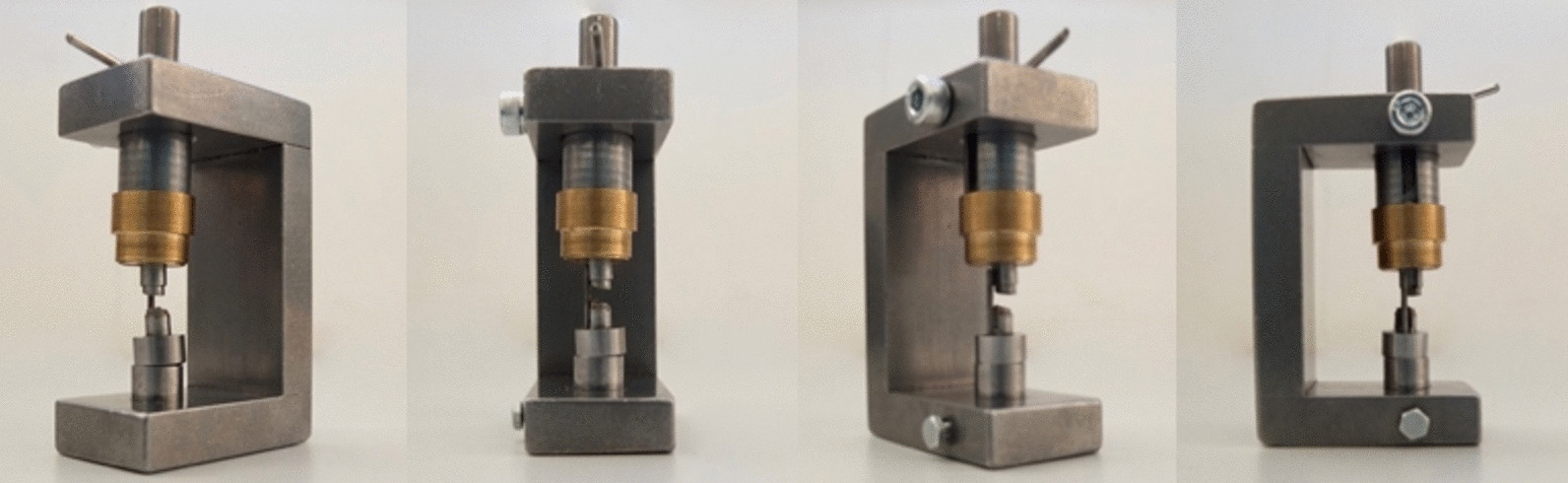
Depiction of the novel vertebral testing device in counterclockwise rotation from left to right starting with a slanted frontal viewpoint and ending with a viewpoint of the left side

**Fig. 2:**
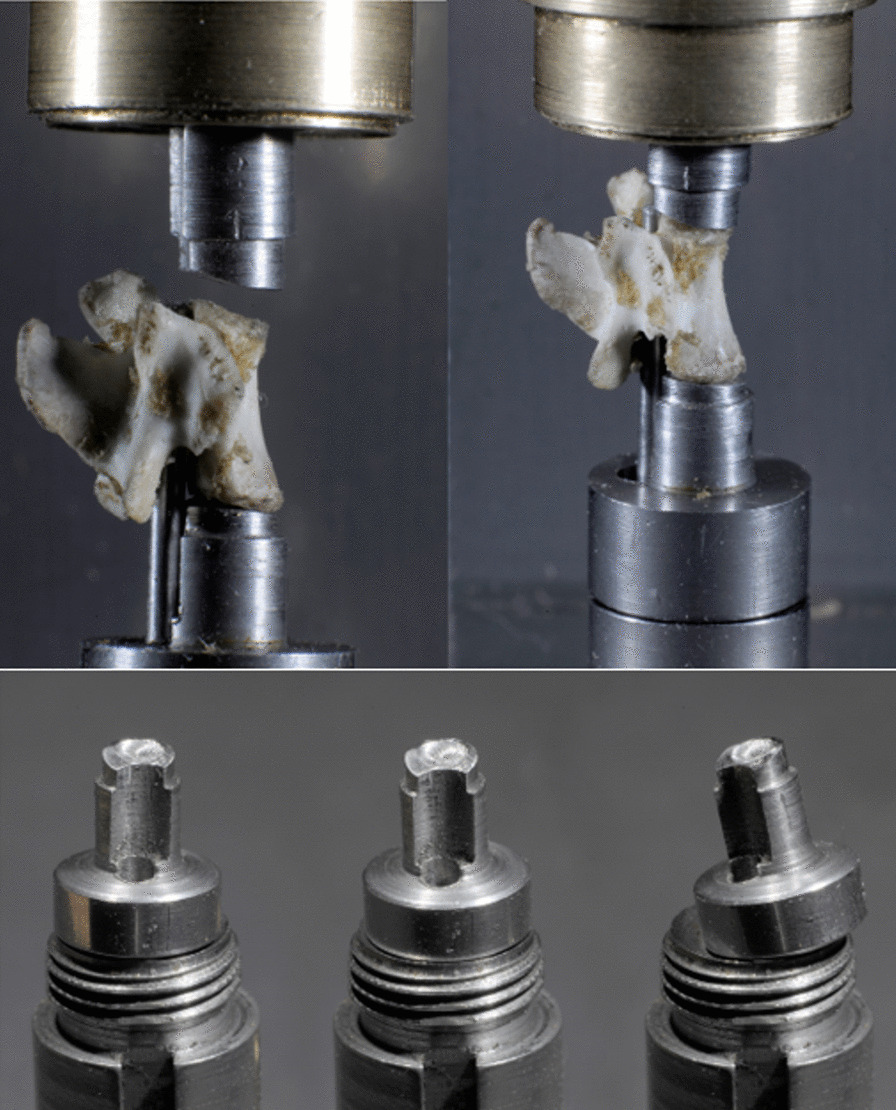
Depiction of the upper and lower stamp of the new biomechanical testing device. Top right and top left image: a test-vertebra mounted in the testing machine with the pin inserted into the vertebral foramen, the base plate placed onto the lower punch and the top punch lowered onto the top plate. Note in the top left image, the natural incline of the vertebra which is addressed by the slanted nature of the top stamp. Lower image: lower punch with lower stamp with an exemplary description of the different ways of adjustment in all directions. Both systems used together ensure a perfect fit for each individual vertebra

**Fig. 3:**
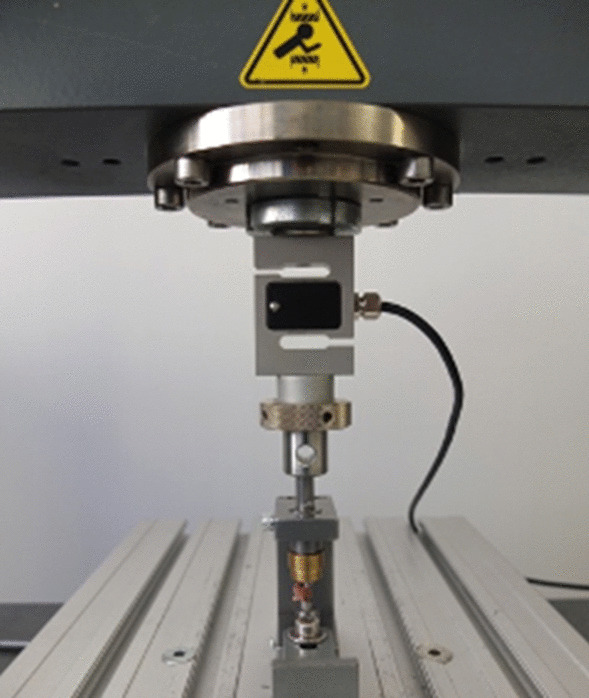
Test set-up. Fixed novel testing device with vertebral body specimen aligned under the Zwick Testing machine

This testing machine consists of a C-shaped, solid, stainless-steel framework, on which an upper and lower punch, with a stamp on each end facing each other, is mounted (Fig. 1). The lower punch is fixed onto the base and has a vertical pin with a diameter of 1 mm, onto which the vertebral body can be placed with the pin inserted into the vertebral foramen, holding the vertebral body in position, simulating the ligamentous fixations in vivo. The upper punch is inserted through a hole in the upper arch of the framework and is held in place by a metal pin. Both the upper and lower punch can be rotated around their own axis. Additionally, the lower punch can perform translational movements to perfectly adjust the inclination according to the vertebra to be tested. The stamp surfaces are designed to be slightly inclined in order to simulate and adjust to the naturally inclined anatomy of the corpus of the vertebra, which displays an inclination going from postero-cranial to antero-caudal in the sagittal axis of the vertebra (Fig. 2). With both stamps slanted and the potential for translational movement of the lower stamp, the inclined surfaces of the stamps and the inclined surfaces of the vertebral bodies can be conformationally aligned. This ensures the precise adjustment of the stamps to the natural shape and orientation of the vertebral body and assumably allows for a force application along the natural vectors including compression, torsion, shear stress and tensile forces similar to those in vivo [13]. After placing the vertebral body into the testing device and adjusting all machine elements to provide for a good conformational fit, the testing device is then placed and fixed below the force application machine. The force application stamp was placed onto the upper punch to transfer the force in an axial fashion. A ZWICK Z020/TND testing machine (ZWICK-/Roell, Ulm, Germany) was used for the mechanical testing of the specimen (Fig. 3). Regarding the load cell, an “S”-shaped force sensor type KAP-S (Fa. AST, Wolznach, Germany) capable of measuring compression as well as traction and a nominal load of 2kN was utilized.

### Mechanical testing procedure

After thawing the lumbar vertebrae for two hours at room temperature, the testing was initiated and the set up conducted as described above. At the start of the test, the distance from the force arm to the punch of the breaking device was approximately 0.5 mm. The test was started after the force had been zeroed. The data were recorded using the “testXpert” software. The machine crossbeam was lowered at a constant speed of 2 mm/min. The changes to the specimen were detected and displayed precisely and at a high frequency. The analog-to-digital converter operated at a sampling rate of 400 kHz and 24-bit resolution over the entire measuring range. Every change in displacement and force is acquired and recorded synchronously in time on all measuring channels. The endpoint of the measurement was defined as either a drop in force over or equal to 50% of the recorded maximum force or a displacement of the force arm by more than 2 mm.

### Data evaluation

**Fig. 4:**
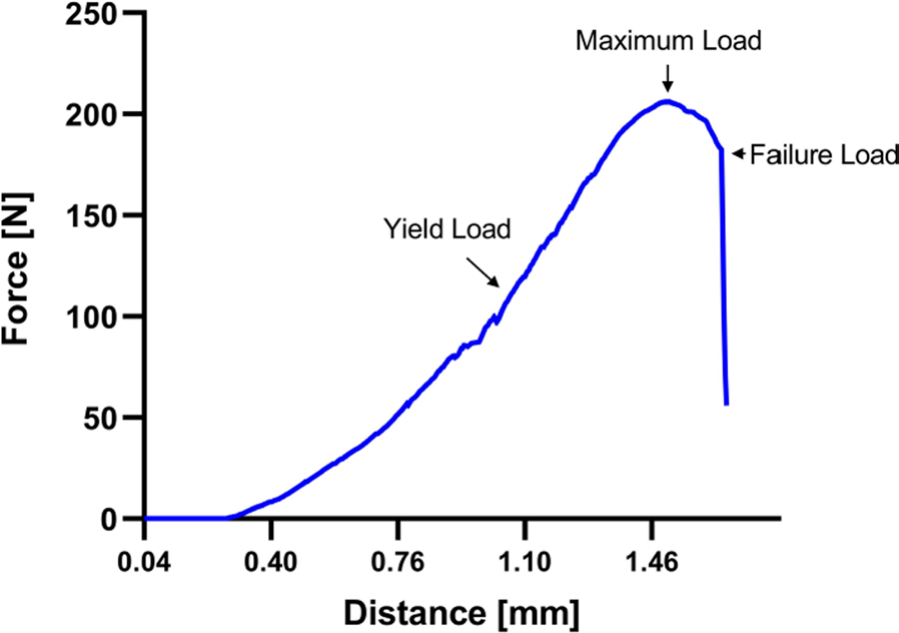
Exemplary graphical visualization of the force recorded during the biomechanical testing procedure. Plotted were the distance travelled by the stamp in millimeters (mm) against the force exerted in Newton (N). Derived from the data were the Stiffness (S), the yield load (yL), the maximum load (mL) and the failure load (fL). The first part, up to the yield load, depicts elastic (reversible) deformation. The second part, from the yield load on, depicts plastic (irreversible) deformation

The forces analyzed in this study correspond to the axial direction of the specimen. The continuously recorded force in Newtons was graphically plotted against the traveled distance of the stamp (Fig. 4). From this graph, the stiffness (S), the yield load (yL), the maximum load (Fmax) and the failure load (fL) were assessed. The term stiffness (S) is the quotient of the generated change in load (δF) and the resulting change in position (δf) also referred to as deformation. The reciprocal value of the stiffness is the compliance (N) [4].

$$S=\frac{\delta F}{\delta f}=\frac{1}{N}$$

From this, it can be deduced that a higher load change (*δF)* has to be exerted on a component with a higher stiffness in order to achieve the same deformation (δf). The stiffness is determined by the slope of the regression line before the elastic limit as the transition between elastic and plastic deformation. The elastic limit is the mechanical stress below which the material displays reversible deformation when the load is removed. When the elastic limit is exceeded, plastic deformation (irreversible deformation) occurs. The point where reversible deformation transits into irreversible deformation is defined as the yield load, which in this study defines the point at which first microfractures occur. The yield load was defined in our study as the decrease in stiffness by two standard deviations. For calculation of the yield load, the regression line and the standard deviation with the individual digital data of the linear part of the graph was used. The maximum load was defined as the largest measured force on the vertebral body in the testing process. The failure load was the force recorded at the point of final bone fracturing.

### Statistics and evaluation

Statistical analysis was undertaken using R (v. 4.0.3) within RStudio (v. 1.3.1093) with the additional package *rstatix*. The parameters to be analyzed (stiffness, yield load, maximum load, failure load) were normally distributed (Shapiro–Wilk test). Therefore, an unpaired t-test was applied (*p* < 0.05) to test for overall differences between groups (non-osteoporotic vs. osteoporotic) after ensuring a homogeneity in variances between groups (F-test). The results were then compared to values found in the literature for the same biomechanical parameters.

## Results

The overall handling of the machine as well as the positioning of the samples were feasible. The fact that the newly designed machine is itself compact with a height of 10 cm, a width of 5 cm and a length of 7 cm, allowed for easy placement into the Zwick testing machine directly underneath the force arm. After positioning the newly designed device into the Zwick testing machine as desired, it was fixed in the correct position underneath the force applicator with screw-tongs to the bottom plate of the Zwick testing machine and could remain in this position for every force application for the rest of the investigation, keeping the position the same for every bone sample. The placement and fixation of the vertebral body onto the lower punch were easily conductible due to the pin, which was placed inside the vertebral foramen. The following fine adjustment of the slanted top and base plates of the vertebrae was, due to the easily adjustable lower punch, easily feasible under visual control. Through the open construction of the machine, all elements were at all times visually controllable, also during the process of the force application.

**Fig. 5:**
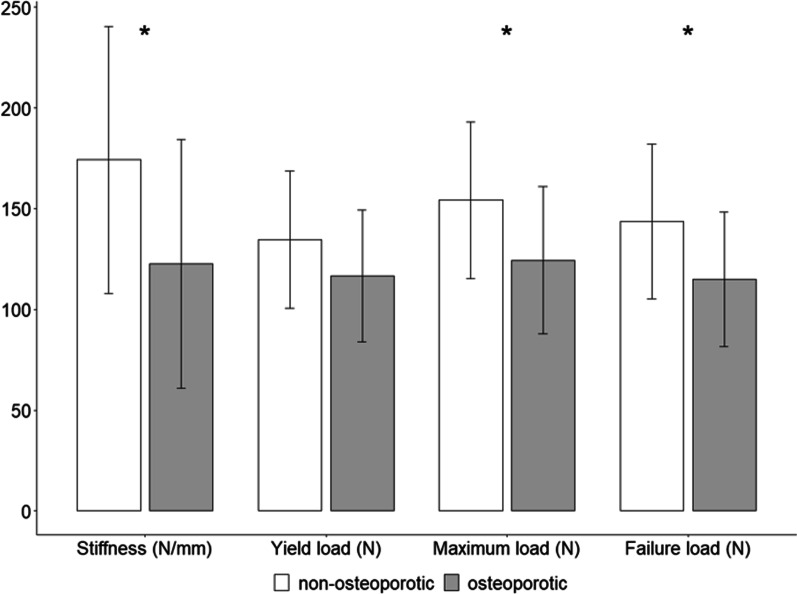
Results for stiffness, yield load, maximum load and failure load of the biomechanical analysis using the newly developed biomechanical testing machine for non-osteoporotic healthy bone (SHAM = Group 1) and osteoporotic bone (OVX = Group 2) in rat lumbar vertebrates. The statistically significant differences are marked with and asterisk (*). N: Newton, mm: millimeter

The stiffness of the vertebral body for Group 1 (non-osteoporotic) was significantly higher (*p* = 0.0167) compared to Group 2 (osteoporotic) with a mean and standard deviation (SD) of 174.2 ± 16.1 N/mm and 133.2 ± 14.0 N/mm, respectively. The yield load for Group 1 was in average 134.6 ± 8.3 N and thus nonsignificantly higher (*p* = 0.1023) than the average yield load for Group 2, which was 125.8 ± 7.0 N. The maximum load for Group 1 (154.2 ± 9.4 N) was in average significantly higher (*p* = 0.0185) than the maximum load for Group 2 (133.5 ± 7.7 N). Finally, the failure load was significantly higher for Group 1 (*p* = 0.0169) with 143.8 ± 9.3 N compared to 123.4 ± 7.0 N for Group 2. These results are presented in Fig. 5 and Table 1.

**Table 1:** Summary of the results with corresponding standard deviation of the different biomechanical parameters investigated regarding the respective group

	Stiffness (N/mm)	Yield load (N)	Maximum load (N)	Failure Load (N)
Group 1 (non-osteoporotic)	174.2 ± 16.1	134.6 ± 8.3	154.2 ± 9.4	143.8 ± 9.3
Group 2 (osteoporotic)	133.2 ± 14.0	125.8 ± 7.0	133.5 ± 7.7	123.4 ± 7.0
*p* value	0.0167	0.1023	0.0185	0.0169

N, Newton; mm, millimeter

A *p* value < 0.05 was considered significant

## Discussion

Bone tissue research is becoming a progressively more interesting and interdisciplinary field of research due to the increasing burden of osteoporotic fractures, including vertebral fractures, as the disease of osteoporosis is becoming more prevalent as the age demographics are changing [10, 11]. An elemental part of this research is the assessment of the biomechanical properties of bone, which are extensively evaluated by various biomechanical testing technologies [1–5].

When biomechanically analyzing bone, different aspects have to be taken into consideration. Bone is a continuously changing tissue, adjusting to stress and load changes by remodeling its trabecular and cortical framework [14]. Further, bone is to be seen as a unit, where trabecular and cortical bone are working together. Thus, evaluating biomechanical properties of only one aspect of the whole unit, by for example only testing a small piece of solely trabecular bone cut out of a whole bone unit (e.g., Yao et al. [8]), will not be representative of the actual biomechanical properties present in living organisms under physiological conditions [8]. Therefore, to receive representative results for biomechanical properties present in bone, especially when researching bone diseases such as osteoporosis, it is vital to be able to test the bone as a whole, three-dimensional unit, simulating physiological conditions as closely as possible. Considering the three-dimensional shape, under physiological conditions the top and base plate of a vertebra receive the applied forces, compression, shear and torsional, to then conduct it onto the trabecular network and the circumferential cortical aspect. Further, the anatomical orientation of the top and base plates of vertebrae is not parallel to the horizontal plane but slanted with an angle, which makes a conformal contact of the complete vertebra surface with a simple horizontally oriented force applying stamp not possible. If this was the case, only the topmost aspect of the vertebral surface would be in contact and receiving the forces, causing a distortion of force distribution. Both aspects are taken into consideration in the newly developed biomechanical testing device, with adjustable top and bottom stamps with angled surfaces that can adjust adequately to the top and base plate angle to distribute the force to the whole vertebra surface as it would be distributed under physiological conditions, to allow the forces to be conducted as close to the in vivo situation as possible. This avoids application of non-anatomic, non-physiological forces and thus prevents distortion of the biomechanical testing results.

Further, a stable fixation needs to be guaranteed for the force application, as the vertebral bodies in living organisms are held in place by a firm fibrous system, spanning especially around the vertebral foramen, preventing dislocation when forces are in effect. This is ensured by using the vertebral foramen as natural biologic structure to secure and align the vertebra within the machine with a pin inserted into the vertebral foramen. During the testing, there was no visible dislocation of any of the vertebrae and no fracture in the area of the pedicles to be observed. Other studies have fixed the vertebrae in other fashions, such as with sandpaper on the bottom base plate to keep the base plate form dislocating during loading in the work of Kraxenberger et al. or by confining the trabecular specimen in a loading chamber, having circumferential contact of the cancellous bone with another material, potentially distorting registered forces due to frictional influence and not considering the anatomical situation of the bone itself [6, 8].

In addition to biomechanically testing the vertebral bodies with a normal metabolic bone situation, this study also investigated the same biomechanical parameters of lumbar rat vertebrae in an osteoporotic bone situation. It is known that in osteoporotic bone, the biomechanical properties are diminished, leading to a weaker and more fracture prone bone structure, also in vertebral bodies [15–17]. Moseklide et al. and Jiang et al. found decreased biomechanical parameters in rat vertebrae in ovariectomized rats using compressive testing, and both authors however used again only a portion of the vertebrae in form of a trabecular cylinder [18, 19]. In this study, it was thus to be expected that the biomechanical properties of the osteoporotic lumbar vertebrae were to be inferior to the biomechanical properties of the non-osteoporotic lumbar vertebrae, which was indeed the case. The biomechanical parameters tested with the new biomechanical testing machine showed a lower value in the osteoporotic setting when compared to the respective non-osteoporotic group in all investigated parameters, with significant differences in stiffness, maximum load and failure load. This shows that the newly designed device is detecting structural changes translated into biomechanical parameters adequately.

It is now crucial to compare the data, assessed by the newly developed device to those retrieved in the literature. Long et al. conducted compression tests also on the fifth lumbar vertebra in a rat model. They removed the vertebral arch and spinous and transverse processes and removed the top and base plate of the vertebrae until two parallel surfaces were achieved. Then the prepared vertebrae were placed on a stainless-steel plate while a second stainless-steel plate was lowered onto the vertebrae and force was applied until bone failure. Here they achieved a maximum load for the SHAM-Group of 150N and 140N for the osteoporotic group, which is very similar to the measurements achieved with the new testing device of this study. The stiffness was for the SHAM-Group at 650 N/mm and for the OVX-Group at 600 N/mm, which was higher than in our experiment. However, it has to be considered that Long et al. altered the vertebrae by removing the top and bottom plate to achieve two parallel surfaces, which can potentially alter the results and does not necessarily mirror the actual physical forces present as they would be in a whole anatomical unit [20]. Morton et al. recorded a higher maximum force and stiffness for the SHAM and the OVX-Group compared to our investigation, however, here the fourth lumbar vertebrae were furnished with PMMA-Adaptors on both the top and base plates. Here the PMMA, according to the pictures of their work, also slightly encompassed the cortical sides of the vertebral body and extend beyond the base and especially top plates, which can therefore potentially distort the actual biomechanical forces recorded and lead to the higher values recorded by Morton et al. [21]. Also, the stiffness for PMMA is less than that for stainless steel, however, by using a PMMA adaptor on which the force is applied through steel plates, there is an additional material entity between the force application stamp and the vertebra. In the newly designed device, the stamps have direct contact with the top and bottom plates, making the application of an extra material superfluous and thus eliminating the influence of a potentially altered force application through materials with different innate properties. This again shows that there is a need for a biomechanical testing machine that takes into account the anatomic design of the vertebra, since in the literature there have been so many different strategies developed, trying to address the anatomical incongruency of the vertebral body. Since there have to our knowledge not been any biomechanical investigations of rat lumbar vertebrae respecting the vertebrae as a three-dimensional functioning whole unit, we suggest our obtained results to be considered as reference values for the field of biomechanical testing of rat vertebrae, since the values correspond to those in the literature.

There are certain limitations to this study. The bone entity investigated with the newly developed machine, is very small, which just in the aspect of its small size is less reliable as for example a sheep model or a human cadaveric model, which are easier in handling and visually adjusting. Also, despite the fact that the technique used was standardized and all vertebrae underwent the same process, one could consider that freezing of the bone, even only for a short time span, might influence the microstructure in the process of freezing and defrosting. It has however been shown that fresh specimen or frozen specimen are superior to other kinds of preservation, for example formaldehyde [7].

## Conclusions

In conclusion, the new biomechanical testing device was designed and developed to evaluate the biomechanical properties of whole lumbar vertebral bodies of a small animal model as a whole, three-dimensional unit since the existing techniques either only test a portion of the vertebrae or fail to consider all anatomic and physiologic aspects of it. By providing an anatomically natural force load application which could respect the anisotropic properties of the vertebral bone, this device may provide results which are less influenced and biased by fixation methods and thus provide more realistic results for the biomechanical properties of whole rat vertebral bodies when compared to an established animal model with and without osteoporosis. Here, the device detected the inferior biomechanical properties in an osteoporotic bone metabolism setting and has achieved similar results as other test protocols in the literature. Therefore, this study suggests that the new biomechanical testing device is suitable for testing the biomechanical properties of small vertebral bodies and giving a more realistic depiction of the biomechanical properties that would be present under physiological conditions by testing the vertebral body as a whole, three-dimensional and functional unit. Thus, it can be used to investigate the biomechanical properties in different study protocols which look at different bone metabolism situations, for example in medication administration, showing the biomechanical situation of the vertebral body as a whole, functional unit.

## Data Availability

The datasets used and/or analyzed during the current study are available from the corresponding author on reasonable request.

## Abbreviations

fL: Failure load
min: Minute
mL: Maximum load
mm: Millimeter
N: Newton
OVX: Ovariectomy/ovariectomized
S: Stiffness
yL: Yield load
